# Oxidatively Modified Proteins in the Serous Subtype of Ovarian Carcinoma

**DOI:** 10.1155/2014/585083

**Published:** 2014-03-27

**Authors:** Sharifeh Mehrabi, Edward E. Partridge, William Seffens, Xuebiao Yao, Felix O. Aikhionbare

**Affiliations:** ^1^Department of Medicine, Morehouse School of Medicine, Atlanta, GA 30310, USA; ^2^Comprehensive Cancer Center University of Alabama at Birmingham, Birmingham, AL 35249, USA; ^3^Department of Physiology, Morehouse School of Medicine, Atlanta, GA 30310, USA

## Abstract

Serous subtype of ovarian cancer is considered to originate from fallopian epithelium mucosa that has been exposed to physiological changes resulting from ovulation. Ovulation influences an increased in inflammation of epithelial ovarian cells as results of constant exposure of cells to ROS. The imbalance between ROS and antioxidant capacities, as well as a disruption of redox signaling, causes a wide range of damage to DNA, proteins, and lipids. This study applied spectrophotometric, dinitrophenylhydrazone (DNPH) assay, two-dimensional gel electrophoresis, and Western blot analyses to assess the levels of oxidatively modified proteins in 100 primary serous epithelial ovarian carcinoma and normal/surrounding tissues. These samples were obtained from 56 Caucasian and 44 African-American patients within the age range of 61 ± 10 years. Analyses showed that the levels of reactive protein carbonyl groups increased as stages progressed to malignancy. Additionally, the levels of protein carbonyls in serous ovarian carcinoma among African Americans are 40% (*P* < 0.05) higher relative to Caucasian at similar advanced stages. Results suggest that oxidative stress is involved in the modification of carbonyl protein groups, leading to increased aggressiveness of epithelial ovarian tumors and may contribute to the disease's invasiveness among African Americans.

## 1. Introduction

Epithelial ovarian cancer (EOC), with its various histological subtypes, is the fifth leading cause of cancer mortality among women in the United States [1]. Serous ovarian carcinoma is the most common and most aggressive subtype of EOCs [2, 3]. For this disease, differences in survival rates between African-American and Caucasian women are substantial, despite advances in surgical and chemotherapeutic management of the disease [1]. Older age and a family history of EOC are risk factors, but the disease etiology and the differences in survival rates in various groups of patients are far from being defined. A difficulty is that multiple genes are involved in the origin and in development of invasive types of EOCs [3, 4].

Ovarian cells, especially epithelial cells, are constantly exposed to ROS [5], which are generated during repeated ovulation and cause inflammation that is considered to be involved in ovarian carcinogenesis [6, 7]. Since most ovarian cancers appear in the surface epithelium, repetitive ovulation is thought to be a causative factor [8, 9]. ROS are involved in the development and progression of many human diseases, including cancer [10]. Oxidative stress is defined as the imbalance between ROS and cellular antioxidative capacities and is based on a disruption of redox signaling [11].

In addition to lipids [12] and DNA [13], proteins are targets for modifications resulting from oxidative stress. In ovarian cystadenocarcinoma, there is an increase in products associated with oxidative stress, such as 8-hydroxy-2′-deoxyguanosine relative to normal ovarian tissues [6]. Oxidized proteins that accumulate during aging are increased with oxidative stress and in some pathological conditions [10, 14]. The most protein modification caused by oxidative stress is the carbonyl groups; the most sensitive amino acids are arginine, lysine, proline, threonine, and glutamic acid. Reactive protein carbonyls reflect the degree of oxidative damage and serve as a biomarker for oxidative stress [15, 16]. For detection of reactive protein carbonyl groups, several methods are available, including 2,4-dinitrophenylhydrazone (DNPH) assay and Western blot immunoassays [17–20]. In the present study, levels of reactive protein carbonyl groups were measured in samples of normal tissue and tissues of early and invasive stages of serous ovarian carcinomas, including samples obtained from Caucasians and African Americans. These experiments were performed to determine the role of oxidative stress during ovarian carcinogenesis and assess the relationship of reactive carbonyl levels with the extent of cancer in the tissues.

## 2. Materials and Methods

### 2.1. Study Samples

One hundred primary epithelial serous ovarian tumor tissues were obtained from the Southern Regional Cooperative Human Tissue Network and the University of Alabama at Birmingham (UAB) Ovarian Spore Center. These samples were stabilized by snap-freezing immediately after excision and dissection. The dissected tissues were placed in cryovials and immersed in liquid nitrogen. All samples were transferred to −80°C for long-term storage as recommended for measurement of proteins with reactive carbonyl groups [21]. Of the tissue samples, 44 were from African Americans and 56 were from Caucasians. The mean age of the patients was 61 ± 10 years. The breakdown of the 100 primary serous epithelial ovarian tissues were 9 control, normal surrounding, and 91 cystadenoma, borderline, carcinoma, and papillary adenocarcinomas (Table 1). All tissue samples were microdissected, diagnosed, and histopathologically confirmed by pathologists. Tumor stages were determined on the basis of criteria outlined by the International Federation of Gynecology and Obstetrics. Demographic characteristics of the patients were grouped based on the clinical diagnosis (Table 1). All studies were implemented under protocols approved by Institutional Review Boards of Morehouse School of Medicine and the University of Alabama at Birmingham.

### 2.2. Extraction of Cytosolic Fractions

The cytosolic fractions of the tissue samples were prepared by differential centrifugation using mitochondria/cytosol fractionation kits (BioVison, CA.). Approximately 400 mg of each sample was cut into the small pieces, placed in a 2 mL plastic tube on ice, and washed twice with ice-cold phosphate-buffered saline (PBS). Each tissue sample was mildly homogenized in an ice-cold Dounce tissue grinder and centrifuged at 700 ×g for 5 min at 4°C. The supernatant was removed, and 1 mL of homogenizing buffer containing protease inhibitors was added. The sample was incubated on ice for 10 min and then homogenized in an ice-cold Dounce tissue grinder, with about 50–60 passes. The homogenate was transferred into a 1.5 mL microcentrifuge tube and centrifuged at 700 ×g for 10 min at 4°C. The supernatant was collected, transferred to a fresh 1.5 mL microcentrifuge tube, and centrifuged at 10,000 ×g for 30 min at 4°C. The supernatant was collected as cytosolic fraction. This fraction was treated with a 1% streptomycin sulfate solution for 15 min to remove DNA, which could react with DNPH and contribute to the reactive carbonyl level of homogenates. After incubation, samples were centrifuged at 13,000 ×g for 15 min at room temperature. The supernatant which contained DNA-free cytosolic fraction was collected and saved for the DNPH assay.

### 2.3. Measurement of Total Protein Concentration

A microplate DC protein assay (BioRad) was used to measure the protein contents of the samples. Each sample was analyzed in duplicate, and a pooled tissue sample was included in each plate to estimate the interassay coefficient of variation and the coefficient of variation, which was determined to be 4.9%. A standard bovine serum albumin (BSA) containing 0.2 mg/mL to 2.0 mg/mL protein was prepared in the same homogenizing buffer and analyzed along with samples. A fraction of 5 *μ*L of each sample and standard protein was added to the well of a 96-well microplate followed by adding 25 *μ*L of reagent A and 200 *μ*L of reagent B as recommended by BioRad protocol. The plate was placed on the plate mixer and mixed for 5 sec, and then it was incubated at room temperature for 15 min. The absorbance at 750 nm was determined spectrophotometrically, the protein concentration of each homogenate was extrapolated from a standard curve. Samples of an extract of MCF7 cells and a protein extract from a control cell line were included in each run as positive controls.

### 2.4. Protein Carbonyl Assay

Oxidized protein modifications in serous ovarian cancer samples were determined by measuring reactive protein carbonyl groups. ROS react with amino acid residues in protein, particularly histidine, arginine, lysine, and proline, to produce carbonyl functions that can react with DNPH, leading to formation of stable dinitrophenylhydrazone adducts [20, 22]. This reaction is used to estimate reactive carbonyl content of proteins in human tissues and body fluid [21]. The protein carbonyl content of the homogenates was determined as follows: DNA-free homogenates of serous ovarian tissue samples (0.5 mL) were placed in each microcentrifuge tube labeled as treated samples and control samples. Two mL of 10 mM DNPH (Sigma) in 2 M HCl was added to the treated sample tubes and two mL of 2 M HCl only was added to the control sample tubes which were incubated on a rotator at room temperature for 1 hr. The hydrazone derivatives were precipitated with 20% (wt/vol) trichloroacetic acid, treated with ethanol/ethyl acetate, 1 : 1  (vol/vol) to remove excess DNPH, and reprecipitated with 10% trichloroacetic acid. The pellet was dissolved in 6 M guanidine hydrochloride. Differences between optical densities of DNPH treated and untreated samples were determined spectrophotometrically at 370 nm. The results were calculated as nmole of DNPH incorporated per mg of protein, as determined from absorptivity using the Bear-Lambert equation and an extinction coefficient 22,000 *µ*M^−1^ cm^−1^. To determine the stability of the samples, protein carbonyls were measured in a pooled tissue sample that was repeatedly frozen and thawed. There was no significant difference in protein carbonyl levels after four freeze-thaw cycles. These results are similar to previously described results for plasma samples [23].

### 2.5. SDS-PAGE and Western Blot Analyses

In this study, to determine the number and relative mass of the DNPH derivatized proteins in the tissue samples, SDS-PAGE and Western blot immunoassays were performed using OxyBlot Protein Oxidation Detection Kits (Millipore). Samples of DNPH-derivatized proteins were resolved on 10% SDS-polyacrylamide gels. As a control an underivatized sample of each cytosolic fraction was run along with DNPH-derivatized samples. The proteins were transferred to PVDF membranes blotted with rabbit anti-DNPH antibody and detected with a superSingal West Pico chemiluminescent substrate. A DNPH-derivatized standard protein was used for estimation of molecular weight. Proteins that were oxidatively modified were identified by their appearance as bands in the lane containing the derivatized sample, but not in the lane containing the control. Blots were quantified using the UN-SCAN-IT automated digitizing system, version 5.1 (Silk Scientific Inc.), and the results were expressed as arbitrary units.

### 2.6. Statistics

Data were analyzed using analysis of variance (ANOVA) and Student's* t*-test.

## 3. Results

**Figure 1:**
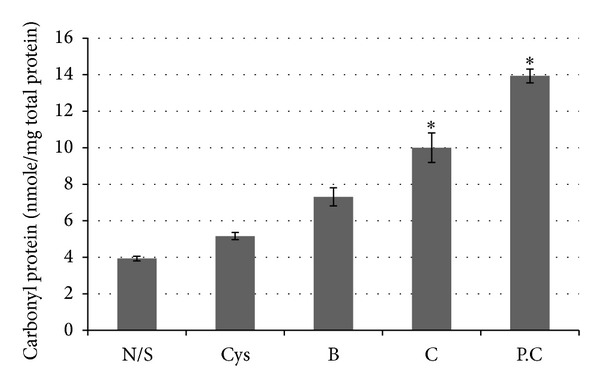
Levels of reactive carbonyl proteins in normal/surrounding and serous subtype ovarian cancer tissues, as measured by the spectrophotometric method. N/S: normal/surrounding; Cys: cystadenoma; B: borderline; C: carcinoma; P.C: papillary carcinoma. Significant differences between groups (*P* < 0.05)* (*N* = 100 samples).

**Figure 2:**
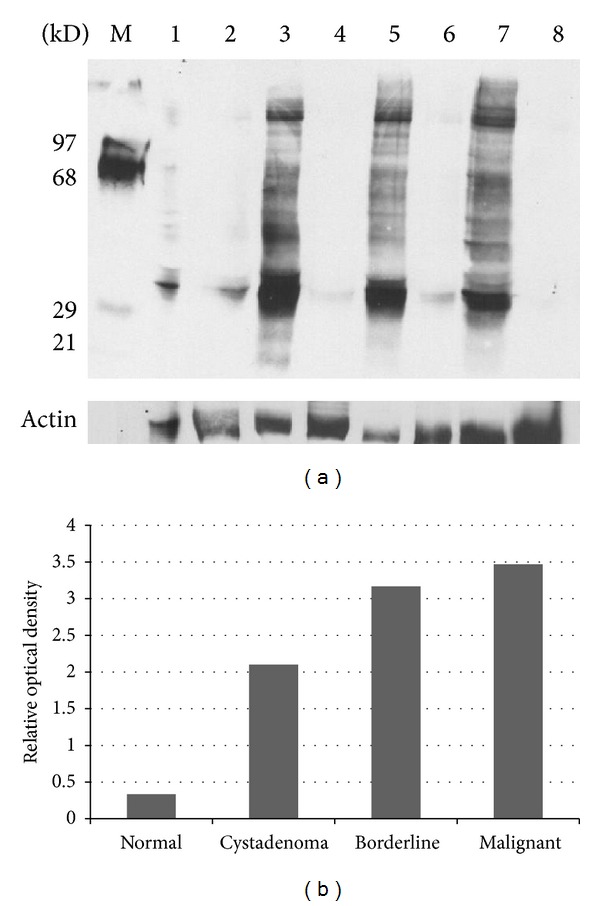
(a) Western blot analysis of expression of reactive carbonyl groups in the cytosolic fraction of serous ovarian carcinoma tissues. Lane M: protein marker; Lane 1: DNPH-derivatized lysate of normal tissue; Lane 2: underivatized lysate of normal tissue; Lane 3: DNPH-derivatized lysate of cystadenoma tissue: Lane 4: underivatized lysate cystadenoma tissue; Lane 5: DNPH-derivatized lysate of borderline tissue; Lane 6: underivatized lysate of borderline tissue; Lane 7: DNPH-derivatized lysate of malignant tissue; Lane 8: underivatized lysate of malignant tissue. (b) The relative optical density on the level of reactive carbonyl proteins using the UN-SCAN-IT system.

*Total Protein Reactive Carbonyls. *In biological systems, free radicals generally lead to oxidative, posttranslational modifications of proteins, a process in which the degree of introduction of reactive carbonyl groups relates to the intensity of the oxidative stress [24]. Proteins from lysates of samples of normal tissues and early and invasive serous ovarian carcinomas were derivatized with DNPH to measure the levels of carbonyl groups by a spectrophotometric method. Progressively increasing levels of carbonyl groups were observed in the derivatized lysates samples from cystadenomas, borderline tissues, carcinoma, and papillary adenocarcinomas (Figure 1). The results of Western blots were similar to those obtained with the spectrophotometric techniques. Relative to normal/surrounding tissues, protein reactive carbonyls were elevated in cystadenomas, borderline tissues, and, notably, the invasive stages (Figures 2(a) and 2(b)).

**Figure 3:**
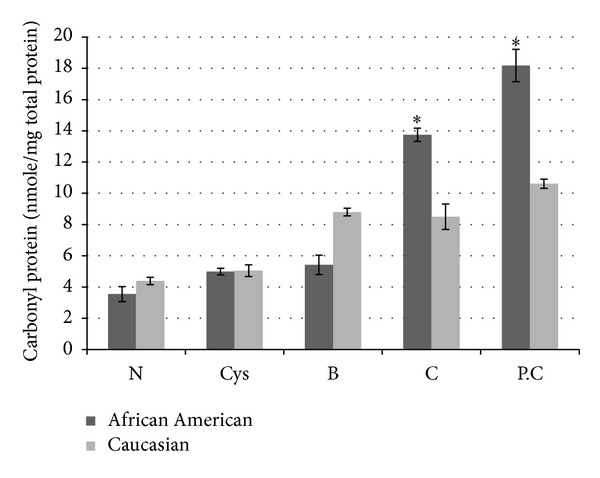
Levels of reactive carbonyl proteins in normal/surrounding and serous subtype ovarian cancer tissues as measured by the spectrophotometric method. N/S: normal/surrounding; Cys: cystadenoma; B: borderline; C: carcinoma; P.C: papillary carcinoma. There were significant differences between samples from African Americans (*N* = 44) and Caucasians (*N* = 56) (*P* < 0.05)*.

**Figure 4:**
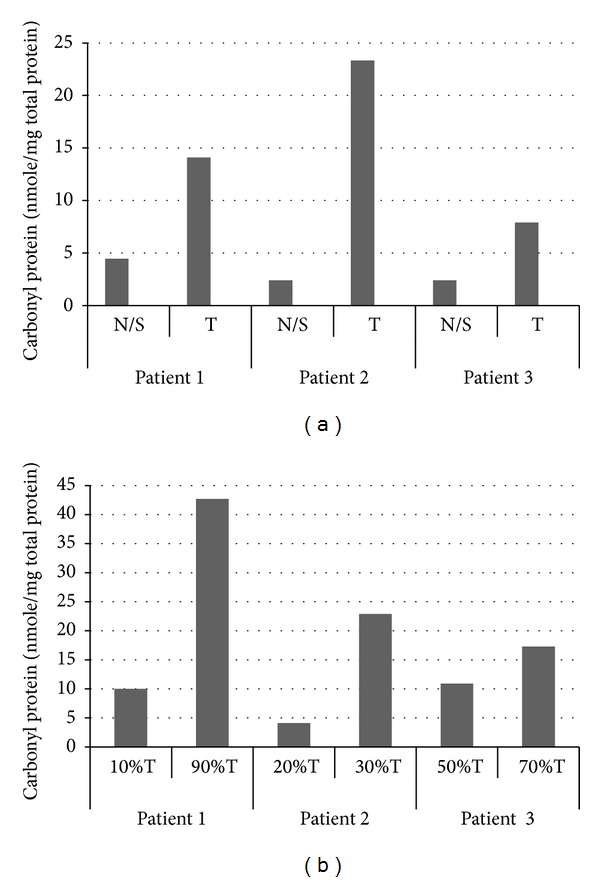
Levels of reactive carbonyl proteins in tumor tissue obtained from different patients as measured by the spectrophotometric method. (a) Levels of carbonyl proteins in normal/surrounding tissues relative to tumor tissues of the same patient (*n* = 3). (b) Levels of carbonyl proteins in tissues with different contents of tumor cells in individual (*n* = 3). N/S: normal/surrounding tissue; T: tumor tissue.

Levels of carbonyl groups were also assessed to evaluate the differences between tissue samples from African Americans and Caucasians (Figure 3). For African Americans, there were, relative to similar samples from Caucasians, 40% lower levels of reactive carbonyls in proteins of borderline tissue samples (*P* < 0.05) and 40% higher levels of reactive carbonyl proteins in carcinomas and papillary carcinoma samples from African American relative to similar samples from Caucasians (*P* < 0.05 for both). In normal tissues and cystadenomas, there were no significant differences between the two groups in levels of protein carbonyls. Differences in the levels of protein carbonyl expression within individual patient samples were also evident. Tissue samples with higher contents of tumor cells exhibited higher levels of oxidized proteins relative to normal/surrounding tissues (Figure 4(a)) and to samples with fewer tumor cells from same patient (Figure 4(b)).

## 4. Discussion

The accumulation of oxidized proteins (protein carbonyls) is associated with the risk of carcinogenesis [9, 14, 15] and age-related diseases [17, 20, 25]. The increase of ROS may result from altered metabolism as well as from inadequate tumor neovascularization. The levels of carbonyl content in tissues have not been determined for the process of ovarian carcinogenesis. Although protein oxidation markers are detected in cells that are already transformed, oxidative modification of proteins may be implicated in the serous ovarian carcinoma subtype, since these cancers are derived from the tubal epithelium of the ovarian surface. Repetitive ovulation is thought to be a causative factor of ovarian cancer [6, 26]. Rupture of follicles involves tissue remodeling, with high cell turnover, characteristic of inflammatory reactions. Oxidative stress is associated with inflammatory processes, resulting from stimuli, such as cytokines (tumor necrosis factor and interleukin-1) and bacterial toxins (lipopolysaccharide) [27]. Particularly in the fallopian tubes, damage to the epithelium resulting from inflammatory responses during ovulation is generally viewed as a secondary event. The primary event is the inflammatory cascade of neutrophil adherence to vascular endothelial cells, disruption of their barrier, and subsequent infiltration of inflammatory cells into the interstitial space, where oxidants and proteases are released and produce mucosal injury. A variety of chronic inflammatory conditions predispose susceptible cells to neoplastic transformation [28]. Inflammatory cytokines, such as TNF-*α* and ROS, activate nuclear factor kappa-B (NF*κ*B) by phosphorylation. In its normal state, NF*κ*B is inhibited by its inhibitory protein (I*κ*B*α*), which downregulates the inflammatory response. In nuclei, NF*κ*B induces the expression of genes involved in cell proliferation, apoptosis, and carcinogenesis [29] and also induces production of proinflammatory cytokines, which enhance the inflammatory responses.

The main effectors in the inflammatory response are ROS. These may directly or indirectly cause damage through their reactions with components of target cells [30]. ROS can also recruit other inflammatory cells, leading to additional ROS production and amplification of damage [31]. Thus, ovulation may be accompanied by inflammation that induces oxidative damage to DNA, proteins, and lipids of the ovarian epithelium [32]. The reactive carbonyl content of protein is the most commonly used marker of protein oxidation [17, 25], and, in cells, oxidized proteins accumulate during aging, accompanied with oxidative stress and some pathological conditions [17–20]. Furthermore, proteins modified by oxidative stress are associated with an increased risk of cancer [17, 33]. In colorectal cancer, there is enhanced oxidative stress relative to normal intestinal tissues [34]. The present results are similar in that they show elevated expression of protein carbonyls in serous ovarian carcinomas relative to normal/surrounding tissue and to cystadenomas. This suggests that oxidative modification of proteins is involved in the formation of ovarian cancers. Carcinogenesis in general may be mediated by oxidative damage to DNA, due to mutations in critical genes, such as the tumor suppressor* p* ^*53*^ [35]. Damage to the* p* ^*53*^ gene may reduce the effectiveness of DNA repair mechanisms and increase the rate of cell division. Cells that are rapidly dividing cells are more prone to errors in DNA replication and repair [26] and may also be more sensitive to oxidative stress, enhancing the risk of carcinogenesis.

As demonstrated here, there are high levels of reactive protein carbonyls in tissue samples of invasive serous ovarian carcinomas from African Americans. The most likely explanation is a racial difference in the intracellular levels of oxidized proteins, reflecting the balance between the rate of protein oxidation and the rate of oxidized protein degradation [36]. This balance is a function of factors leading to the generation of ROS [37]. Various physiological and environmental processes may lead to the formation of ROS and be factors in determining the concentrations and/or activities of the proteases that degrade oxidatively damaged proteins [17]. Such degradation is also dependent upon numerous variables, including the concentrations of proteases that preferentially degrade oxidized proteins, and upon cellular components, such as metal ions, inhibitors, activators, and regulatory proteins, that affect their proteolytic activities. For example, oxidized forms of some proteins, for example, cross-linked proteins [38–40] and proteins modified by glycation [41] or by lipid peroxidation products [42], are resistant to proteolysis and could lead to production of protease inhibitors that hinder degradation of the oxidized forms [38, 42]. Therefore, inactivation of these protein inhibitors could enhance the action of proteases, such as elastase, plasminogen activator, and plasmin. This process could facilitate tumor invasion and metastasis [43], particularly in various individuals or subgroups.

A reduced dietary intake of antioxidants and an impaired mitochondrial function may render African Americans more vulnerable to diseases associated with oxidative stress [44]. This concept is based on results obtained from a study of racial differences in association of oxidative stress and insulin sensitivity in African- and European-American women. Therefore, we speculate that the effect of the elevated levels of protein carbonyls in ovarian cancer tissues from African-American patients may be involved in the aggressiveness of the disease. However, the current results do not provide evidence that an increase in protein carbonyls is solely the cause of racial differences between African Americans and Caucasians regarding ovarian cancer aggressiveness. Alterations of the redox balance within the cell, leading to oxidative damage to proteins, lipids, and nucleic acids involvement, cannot be ruled out as a cause of this difference.

To our knowledge, this is the first report to demonstrate a relationship between elevated levels of reactive protein carbonyls and the serous ovarian carcinoma subtype and to note differences in expression of reactive protein carbonyls between African-American and Caucasian women bearing the disease. This is noteworthy, since oxidative stress is considered to be triggered by ovulation-induced inflammation. Inflammation normally leads to production of oxidants to kill pathogens, but these oxidants can cause damage to DNA, proteins, and lipids and may be, therefore, involved in ovarian carcinogenesis [26].

A strength of these findings is the similarity of the results for Western blot and spectrophotometric techniques in measuring levels of protein reactive carbonyls in tissue samples of ovarian cancer. A limitation is the relatively small sample size, related to the cross-sectional nature of the study and to the limited population of African-American and Caucasian women with ovarian cancer. To fully understand the contribution of oxidative stress to ovarian carcinogenesis and racial disparities in the aggressiveness of the disease, future research should include a larger sample of women of different ethnic backgrounds and potential involvement of epigenetic regulations such as microRNAs in the regulatory circuitry underlying disparity [45–47].

In conclusion, results from this study demonstrate an association of elevated levels of reactive protein carbonyls, formed by oxidative stress, with serous ovarian carcinogenesis. The results also indicate a racial difference in levels of these carbonyl groups and invasive stages of serous ovarian carcinoma among women bearing this disease. Whether the higher prevalence of aggressiveness ovarian carcinomas in African-American women correlates with greater oxidative damage within these patients deserves further research.

## Figures and Tables

**Table 1:** Characteristics of tumors and patients.

Characteristics	Category	Subcategory	*n* (%)
Ethnicity	African American		48 (48%)
	Caucasian		52 (52%)

Age (years)	≥61	African American	19 (40%)
		Caucasian	29 (60%)
		Total	**48 **
	<61	African American	29 (56%)
		Caucasian	23 (44%)
		Total	**52 **

Differentiation	Normal/surrounding		9 (9%)
	Cystadenoma		12 (12%)
	Borderline		8 (8%)
	Carcinoma		24 (24%)
	Papillary carcinoma		47 (47%)
